# Manganese(II) oxidation by *Bacillus wiedmannii* DX3 isolated from electrolytic manganese residue: Mn(III) as an intermediate and characterisation of biogenic manganese oxides

**DOI:** 10.3389/fmicb.2026.1910017

**Published:** 2026-10-13

**Authors:** Kai Zhong, Qian Zhang, Siting Lu, Juanjuan Yin, Yan Liao, Minghua Gu, Chaolan Zhang

**Affiliations:** 1 College of Agriculture, Guangxi University, Nanning, China; 2 School of Resource, Environment, and Materials, Guangxi University, Nanning, China

**Keywords:** Bacillus, biogenic manganese oxide, Mn remediation, Mn(II)-oxidising bacteria, Mn(III) intermediate

## Abstract

Manganese contamination in soil poses risks to plant growth and human health. In this study, we isolated the manganese-oxidising bacterium *Bacillus wiedmannii* DX3 from electrolytic manganese residue and investigated its Mn(II) oxidation characteristics. The strain exhibited efficient Mn(II) oxidation at different initial Mn(II) concentrations, with a maximum biogenic manganese oxide (BMO) production of 166.89 μM achieved at 5.0 mM Mn(II). Spectroscopic analysis revealed that Mn(III) serves as a key intermediate during microbial Mn(II) oxidation. The biogenic manganese oxides were further characterised using transmission electron microscopy (TEM), X-ray diffractometry (XRD), and X-ray photoelectron spectroscopy (XPS), providing insights into their structural and functional properties. Overall, this study expands our understanding of manganese-oxidising bacteria and provides a mechanistic basis for the potential application of strain DX3 in the bioremediation of manganese-contaminated environments. The biogenic manganese oxides produced through the Mn(II) → Mn(III) → Mn(IV) pathway act as adsorbents for toxic metals and as oxidants for redox-sensitive contaminants. However, their efficacy in real soil and water matrices, as well as the biosafety of this *B. wiedmannii* strain, requires further evaluation.

## 1 Introduction

Manganese is one of the essential micronutrients for plants and an important component of chloroplasts, playing a crucial role in the photosynthesis process (Housh et al., 2022). However, excess concentrations of manganese are harmful to plant growth, resulting in leaf chlorosis, failure to thrive, and plant death (Yang et al., 2022). Due to anthropogenic activities and manganese ore mining, farmland soils adjacent to manganese mines have been found to be contaminated with manganese in China. The toxicity of manganese to plants depends mainly on its availability. The form of manganese available to plants is divalent manganese [Mn(II)], which can be transformed into a stable manganese oxide state through oxidation (Feng et al., 2024; Liu et al., 2023). Manganese-oxidising bacteria are widely present in the environment and are believed to play a major role in the formation of manganese oxides. In addition, a lower soil pH could accelerate Mn(II) dissolution from stable manganese oxides (Etabo et al., 2024). Controlling the dissolution of Mn(II) and promoting Mn(II) oxidation to stable manganese oxides are the main strategies. Owing to the relatively high chemical potential, the rate of manganese oxidised by oxygen in nature is extremely low and is approximately 1,000-fold higher when mediated by microorganisms (Shoiful et al., 2024; Miyata et al., 2024). Therefore, accelerating the formation of manganese oxides by utilising manganese-oxidising bacteria is a promising remediation method for manganese-contaminated soil.

Over the past few decades, many bacteria exhibiting manganese-oxidising activity have been identified and are considered potential microbial resources for soil manganese remediation (Ma et al., 2023). These bacteria have been found in various habitats, including terrestrial and marine environments, where they play important roles in regulating the Mn cycle and the redox cycle of other elements. *Bacillus* sp. SG-1, *Pseudomonas putida* MnB1, and *Leptothrix discophora* strain SS-1 are major typical strains that have been extensively studied for manganese-oxidising activity (Francis et al., 2002). Isolated from a marine environment, *Bacillus* sp. SG-1 oxidises Mn(II) through its spores, whereas the cells are not involved in the oxidising process (Bargar et al., 2000). *Pseudomonas putida* MnB1 is widely distributed in freshwater and soil environments. The oxidation of Mn(II) takes place on the outer membrane by a polysaccharide–protein complex, forming manganese oxides that precipitate on the cell surface (Tran et al., 2018). *Leptothrix discophora* can be found in freshwater environments and exhibits manganese-oxidising activity under both aerobic and anaerobic conditions. Mn(II) is oxidised extracellularly by *Leptothrix discophora*, a process mediated by EPS (Babar et al., 2022; Wu et al., 2022). Although the Mn(II)-oxidising mechanisms of the major model strains have been elucidated in detail, it remains unclear how Mn(II) is oxidised by *Bacillus wiedmannii*.

Soluble Mn(III) is an unstable form of manganese and is considered an important intermediate during microbial manganese oxidation. Li et al. (2024) found that the addition of sodium pyrophosphate (PP), which has a strong complexing affinity for Mn(III), forming stable Mn(III)-PP ligands, could inhibit the biodegradation of oxytetracycline mediated by the manganese-oxidising bacterium *Bacillus brevis*. Furthermore, Min et al. (2022) identified the role of Mn(III) as an intermediate in Mn(II) oxidation by *Shewanella putrefaciens* CN32, contributing to the formation of manganese oxides. To date, it remains unclear whether Mn(III) acts as an intermediate in the Mn(II) oxidation process, which is mediated by *Bacillus wiedmannii*, and the microbial mechanisms for the transformation of generated Mn(III) into manganese oxides are still unclear.

In this study, we isolated a manganese-oxidising *Bacillus wiedmannii* strain from electrolytic manganese residue. The role of Mn(III) during the Mn(II) oxidation process was also analysed. The resulting biogenic manganese oxides (BMOs) were characterised. The results are expected to elucidate the manganese-oxidising characteristics of *Bacillus wiedmannii* and unveil a novel microbial mechanism for Mn(II) oxidation driven by *Bacillus wiedmannii.* These findings highlight the new role of *Bacillus wiedmannii* as a Mn(II) redox driver in promoting manganese immobilisation in soils and sediments.

## 2 Materials and methods

### 2.1 Isolation of Mn-oxidising bacteria

The DX3 strain was isolated from a reservoir of electrolytic manganese residue, which poses a threat to the surrounding agricultural soils. First, 1 g of electrolytic manganese residue was added to a 250 mL Erlenmeyer flask containing 100 mL of sterile LB medium. After incubation at 30 °C with shaking at 200 rpm for 24 h, 1 mL of the suspension was transferred to fresh sterile LB medium containing 50 mM MnCl_2_ and incubated at 30 °C with shaking at 200 rpm for 48 h. After incubation, the dark-brown suspension was serially diluted with sterile distilled water to 10^−6^–10^−8^. Then, 100 μL of each diluted suspension was spread onto LB agar plates containing 50 mM MnCl_2_. The plates were incubated at 30 °C for 36 h. After incubation, colonies with Mn(II)-oxidising activity appeared dark brown in colour. The selected colony was further purified by lineation and designated as DX3. DX3 was incubated in PYCM medium with 50 mM MnCl_2_ at 30 °C with shaking at 200 rpm for 3 d. The leucoberbelin blue (LBB) solution turned dark blue after being mixed with the DX3 culture suspension, indicating that biogenic manganese oxides were generated after the incubation.

For long-term storage of the isolated DX3 strain, a glycerol-freezing method was applied. A single colony of strain DX3 was picked from an LB agar plate and grown overnight in fresh sterile LB medium at 30 °C with shaking at 180 rpm. The overnight culture was then diluted into fresh LB medium and incubated at 30 °C to the exponential phase. Then, 0.8 mL of sterile glycerol (50%) and 1.2 mL of the strain culture were added to a cryotube and thoroughly mixed. The final concentration of glycerol was 20% (v/v). The cryotube was frozen in liquid nitrogen and then stored at −80 °C in a refrigerator.

### 2.2 Whole-genome sequencing of strain DX3 and identification of DX3

Strain DX3 was identified at the species level using whole-genome sequencing. The draft genome was assembled from Illumina paired-end reads (2 × 150 bp) using SPAdes, yielding a genome of approximately 5.4 Mb with a GC content of 35.3%. Average nucleotide identity (ANI) and digital DNA–DNA hybridisation (dDDH) values were calculated against the type strains of closely related species using FastANI and the GGDC 3.0 server, respectively. The highest ANI (99.0%) and dDDH (93.8%) values were obtained against the type strain of *Bacillus wiedmannii*, both exceeding the species-level cut-offs (ANI > 95% and dDDH > 70%). Whole-genome phylogenetic analysis confirmed the clustering of strain DX3 with *B. wiedmannii*. Strain DX3 was therefore assigned to *Bacillus wiedmannii*.

### 2.3 Manganese oxidation assays

The DX3 glycerol stock was thawed and initially streaked onto an LB agar plate, followed by lineation for 12 h. Then, a single colony was picked and transferred into fresh LB medium for an additional 12 h of incubation. The cells were harvested by centrifugation (5,000 rpm, 5 min) and resuspended in the sterile saline solution (0.9% NaCl). The optical density of the cell suspension at 600 nm (OD_600_) was adjusted to 1.0 using the sterile saline solution. Subsequently, 0.2 mL of the cell suspension was inoculated into 20 mL of sterile PYCM medium containing different concentrations of MnCl_2_ (0.5, 1, 2, and 5 mM). The cultures were incubated at 30 °C with shaking at 200 rpm under aerobic conditions for the specified incubation period. The MnCl_2_ solution was filtered through a sterile 0.22 μm membrane before being added to the medium. The suspension was collected for the determination of manganese oxide formation and soluble manganese concentrations.

### 2.4 Preparation and characterisation of biogenic manganese oxides

Strain DX3 was incubated aerobically in PYCM medium containing 5 mM MnCl_2_ at 30 °C with shaking at 200 rpm for 7 d. The biogenic manganese oxides (BMOs) formed were collected by centrifugation (8,000 rpm, 5 min) and purified using the phenol-chloroform-isoamyl alcohol method. The purified BMOs were freeze-dried and stored at room temperature in a zip-lock bag before further use.

The morphology of the BMO sample was determined by transmission electron microscopy (TEM) using an FEI Tecnai G2 F20 microscope (FEI, Hillsboro, OR, USA). X-ray diffractometry (XRD) patterns were collected using a Bruker D8 Advance diffractometer (Bruker, Karlsruhe, Germany) with Cu Kα radiation (λ = 1.5406 Å). Phase identification was carried out by comparing the observed reflections with the ICDD PDF database. The manganese dioxide (MnO_2_; PDF #43–1,455), rhodochrosite (MnCO_3_; PDF #86–0172), and bixbyite-type manganese oxide (Mn_2_O_3_; PDF #41–1,442) phases were identified on the basis of peak positions. As biogenic manganese oxides are typically poorly crystalline, the reflections were broad and of low intensity. Accordingly, the assignment of the dominant MnO_2_ phase is considered robust, whereas the assignments of Mn_2_O_3_ and MnCO_3_ are tentative and represent the most plausible minor phases consistent with the observed reflections. X-ray photoelectron spectroscopy (XPS) was used to analyse the chemical components on the surface of BMOs.

### 2.5 Soluble Mn(III) determination and the Mn(III) trapping assay

Soluble Mn(III) in the culture medium was determined by UV–visible spectrophotometry after complexation with sodium pyrophosphate (PP), which stabilises Mn(III) as a soluble Mn(III)–PP complex (Liao et al., 2024). Briefly, 1 mL of the culture suspension was mixed with 1 mL of the 5 mM PP solution and allowed to stand in the dark for 30 min. After centrifugation at 8000 rpm for 5 min, the supernatant was transferred to a quartz cuvette, and the absorbance was measured at 258 nm using a UV–visible spectrophotometer. The Mn(III)–PP complex exhibits strong absorbance at 258 nm (ε = 6,750 M^−1^ cm^−1^) (Webb et al., 2005). The concentration of solid manganese oxides (i.e., the Mn(III)/Mn(IV) oxide fraction, referred to as biogenic manganese oxides, BMOs) was determined using the LBB assay. The residual soluble Mn(II) concentration was then calculated by mass balance:


$$\begin{array}{l} {\left[\mathrm{Mn}(\mathrm{II})\right]}_{\text{residual}}={\left[\mathrm{Mn}(\mathrm{II})\right]}_{\text{initial}}\\ -{\left[\mathrm{Mn}(\mathrm{III})\right]}_{\text{soluble}}-{\left[\mathrm{Mn}(\text{oxide})\right]}_{\text{solid}} \end{array}$$

Where [Mn(II)]_initial_ is the initial concentration of Mn(II) added to the culture medium; [Mn(III)]_soluble_ is the concentration of soluble Mn(III) stabilised as the Mn(III)–PP complex; and [Mn(oxide)]_solid_ is the concentration of Mn in the solid oxide fraction (BMOs). All terms are expressed in mM. Mn(II) adsorbed onto the cell surface or accumulated intracellularly remains in the Mn(II) pool and is therefore included in [Mn(II)]_residual_.

To investigate the role of Mn(III) in Mn(II) oxidation by strain DX3 and to confirm the oxidation pathway of Mn(II), PP was employed as an Mn(III)-trapping reagent. The PP stock solution was sterilised by filtration through a 0.22 μm filter and added to sterile PYCM medium containing 5 mM MnCl_2_ to achieve a final concentration of 10 mM. Then, 1% (v/v) of the cell suspension was inoculated into the medium and incubated aerobically at 30 °C with shaking at 200 rpm. After incubation, the sample was collected and divided into two aliquots. One aliquot was centrifuged at 8000 rpm for 5 min, and the absorption spectrum of the supernatant was recorded using a UV–visible spectrophotometer. The other aliquot was used for BMO concentration determination using the LBB assay (Francis et al., 2001).

### 2.6 Analysis of the Mn distribution in the cells of DX3

To investigate the Mn distribution in DX3 cells, a sequential extraction method was employed (Wang et al., 2025). A cell suspension of DX3 (OD_600_ = 1) was added to sterile PYG medium containing 0.5, 1, 2, or 5 mM Mn at a 1% ratio (v/v) and incubated at 30 °C with shaking at 150 rpm for 24 h and 72 h. After incubation, 5 mL of the culture was collected in a centrifuge tube and centrifuged at 8000 rpm for 5 min. The supernatant was filtered through a 0.22 μm membrane and marked as residual Mn (F1). Then, the cells were suspended in 5 mL of 50 mM CuSO_4_ and shaken for 8 h. The centrifuged supernatant was filtered through a 0.22 μm membrane and marked as extracellularly adsorbed Mn (F2). The cells were then disrupted by ultrasonication in an ice bath using a cycle of 5 s of operation followed by a 5 s pause for a total duration of 5 min. The disrupted cells were resuspended in 5 mL of 50 mM CuSO_4_ and shaken for 8 h. The centrifuged supernatant was filtered through a 0.22 μm membrane and marked as intracellularly adsorbed Mn (F3). Finally, 5 mL of 20 mM hydroxylamine hydrochloride was added to reduce the manganese oxides. The centrifuged supernatant was filtered through a 0.22 μm membrane and marked as oxidised Mn (F4).

### 2.7 Determination of ETSA and ROS

Electron transfer activity was determined using the formazan (INF) spectrometry method (Liu et al., 2018). Briefly, 5 mL of the strain DX3 culture at 24 h, 48 h, and 72 h, with initial Mn concentrations of 0, 1, 2, and 5 mM, was added to a 10 mL centrifuge tube. Then, the cells were harvested by centrifugation (10,000 rpm, 10 min, 4 °C). After harvest, the cells were washed twice with pre-cooled phosphate-buffered saline (PBS). Then, the cells were resuspended in pre-cooled PBS and adjusted to an OD_600_ of 0.1. Subsequently, 1 mL of the suspension was mixed with 100 μL of 0.5% 2-(p-iodophenyl)-(p-nitrophenyl)-5-phenyl tetrazolium chloride (INT) solution. The mixture was incubated in the dark for 20 min. The enzyme reaction was then terminated by adding 100 μL of formaldehyde, followed by centrifugation at 10000 rpm for 10 min. Then, 4 mL of 96% methanol was added to extract INF from the cells. The absorbance of the extract was measured at 490 nm using a UV–visible spectrophotometer. The protein content of the cell suspension was determined using the Coomassie brilliant blue method.

Reactive oxygen species (ROS) generated in the DX3 culture with different initial Mn concentrations were determined at 24 h and 72 h. The production of ·OH was determined using a fluorescence probing technique with coumarin (Náfrádi et al., 2020). The production of O₂^−^· was determined based on the formation of XTT (2,3-Bis(2-methoxy-4-nitro-5-sulfophenyl)-2H-tetrazolium-5-carboxanilide) formazan (Ai et al., 2024). The concentration of H₂O₂ was determined using the iodine method (Domergue et al., 2023).

## 3 Results

### 3.1 Isolation, purification, and identification of strain DX3

During the isolation and purification process, strain DX3 showed the highest manganese-oxidising activity and the greatest biogenic manganese oxide production, turning the colour of the LBB solution from light blue to dark blue (Figure 1A). After 48 h of incubation, the colonies turned brown (Figure 1B) and developed rough surfaces and irregular edges, which were attributed to the production of manganese oxides and spores. On LB plates supplemented with Mn(II), the medium turned dark brown within 48 h, and the LBB solution on the plate became dark blue (Figure 1C), confirming the manganese-oxidising ability of strain DX3. From the microscope photos, it can be seen that the bacterial cells were short and rod-shaped and stained purple, indicating that the strain was a Gram-positive bacterium (Supplementary Figure S1). According to the results of whole-genome sequencing similarity and phylogenetic analysis, the strain showed a close affiliation with *Bacillus wiedmannii* (Figure 1D). Based on the results obtained above, we named the isolated strain *Bacillus wiedmannii* DX3.

**Figure 1:**
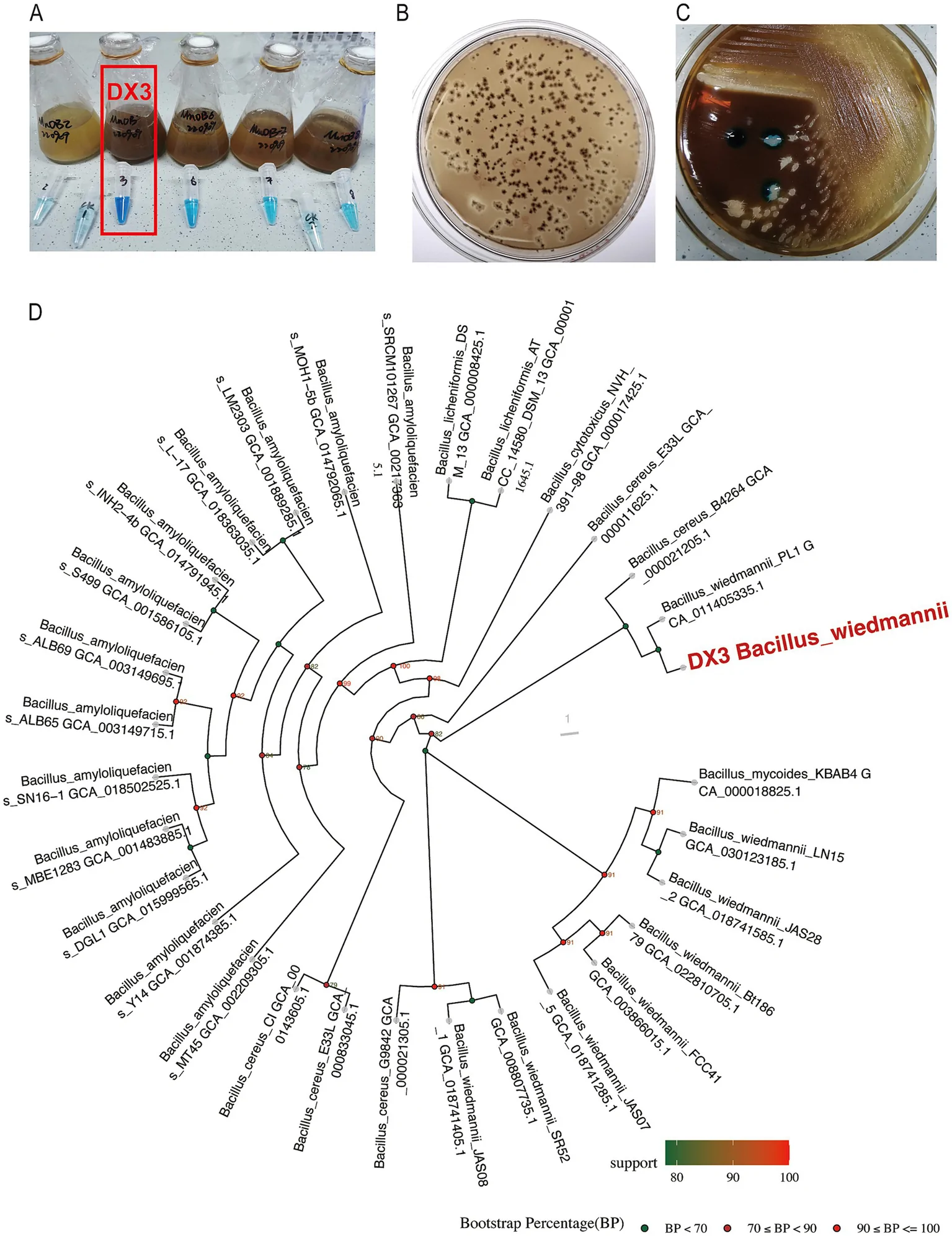
Isolation and identification of manganese-oxidising strain DX3. Cultures of different strains after 48 h of incubation **(A)**. Colonies of the manganese-oxidising strain on the LB plate containing 20 mM Mn2+ after 48 h of incubation **(B)**. Colonies of strain DX3 on the LB plate containing 20 mM Mn2+ after 48 h of incubation **(C)**. Phylogenetic tree of strain DX3 **(D)**.

### 3.2 Growth and manganese oxidative characteristics of strain DX3

To investigate the growth characteristics of strain DX3, a growth curve was plotted under culture conditions without Mn(II) addition (Figure 2A). Upon inoculation into the medium, strain DX3 exhibited a relatively high growth rate and entered the logarithmic phase between 2 h and 16 h. After 16 h, the strain entered the stationary growth phase due to nutrient depletion in the medium. Growth then gradually slowed, and after 22 h, the strain entered the decline phase. Although initial Mn concentrations higher than 5 mM inhibited bacterial growth to some extent, the OD600 values of the media with 5 mM and 10 mM initial Mn concentrations reached levels comparable to those of the 0, 1, and 2 mM groups at 36 h. During cultivation, the pH of the medium gradually increased as the bacteria grew, eventually reaching approximately 8.5 in the absence of Mn(II) (Figure 2B), indicating that strain DX3 can alkalinise the culture medium. Despite the pH reaching ~8.5 at 48 h of cultivation, the distribution diagram of Mn(II) as a function of pH indicates that Mn(II) in the culture medium remains in its ionic form under these conditions (Supplementary Figure S4).

**Figure 2:**
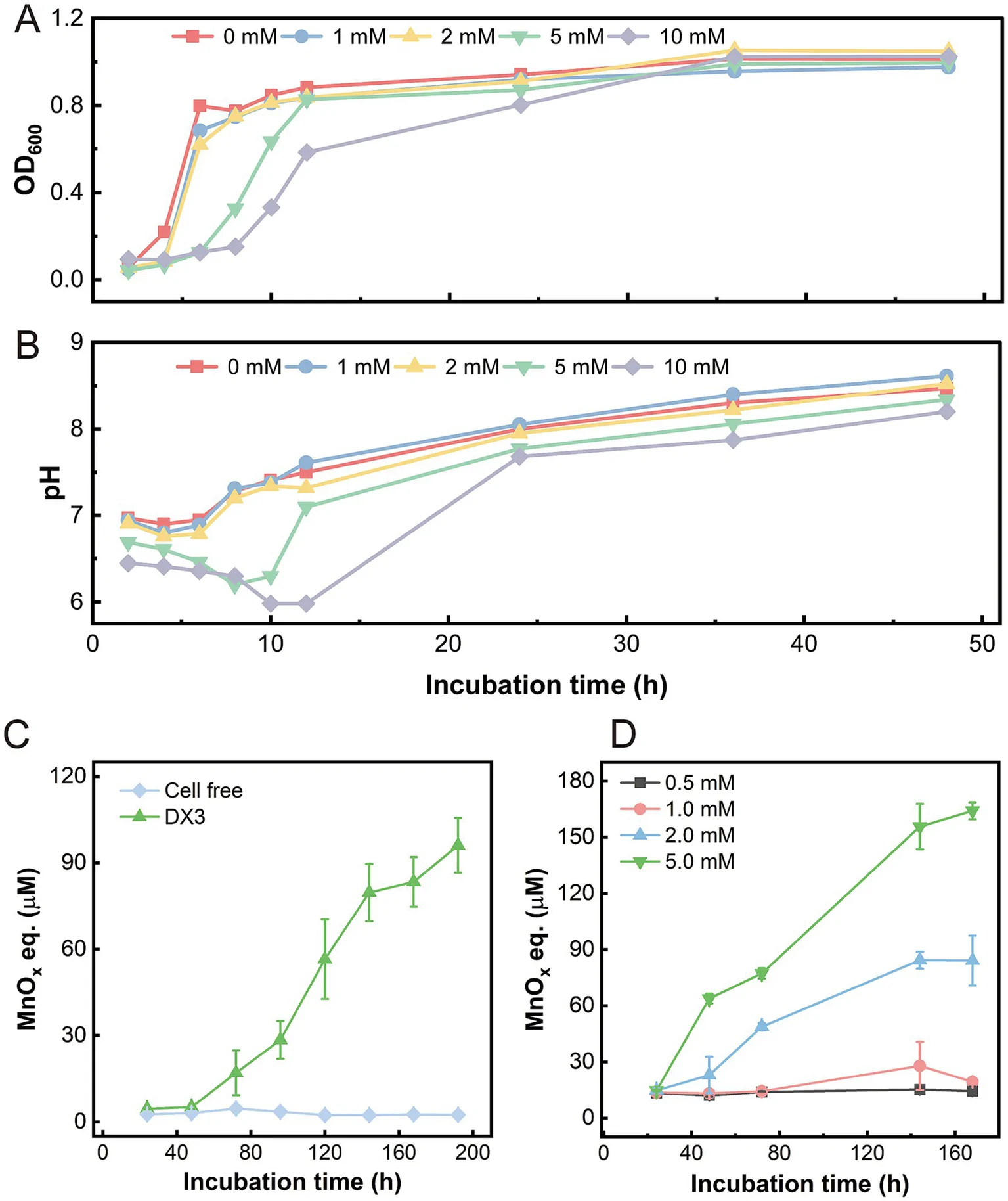
Growth curve of strain DX3 **(A)**. Changes in culture pH during the growth of strain DX3 **(B)**. Manganese oxide generation by strain DX3 incubated in medium with or without Mn^2+^ addition **(C)**. Manganese oxide generation at different initial Mn concentrations during the cultivation of strain DX3 **(D)**. Data are presented as mean ± SD (n = 3).

Batch experiments were conducted to evaluate the manganese-oxidising ability of strain DX3. When strain DX3 was cultured with Mn(II) supplementation, manganese oxides were formed, and the amount of manganese oxides increased both with incubation time and with increasing initial Mn(II) concentrations (Figure 2C). No manganese oxides were detected in the absence of strain DX3, indicating that Mn(II) oxidation was mediated by strain DX3, while abiotic oxidation by oxygen was negligible. Furthermore, no detectable manganese oxides were generated when the initial Mn(II) concentration was 0.5 mM or 1.0 mM (Figure 2D). The concentration of manganese oxides increased slowly during the first 48 h, followed by a sharp increase, reaching a maximum of 166.89 μM after 168 h of incubation in the medium supplemented with 5.0 mM Mn(II), as determined by the leucoberbelin blue (LBB) method, which specifically detects the Mn(III)/Mn(IV) oxide fraction. During the cultivation of strain DX3, the pH of the medium gradually increased and stabilised at approximately 8.3 in the presence of Mn(II). This indicates that alkaline metabolites were produced during the growth and metabolic processes of strain DX3.

### 3.3 Subcellular localisation of bacterial manganese oxidation

To investigate the distribution of Mn inside and outside bacterial cells and the changes in Mn speciation during Mn(II) oxidation by strain DX3, the concentrations of Mn in the residual soluble, extracellularly adsorbed, intracellularly adsorbed, and oxidised fractions were determined by atomic absorption spectrometry (AAS) after sequential chemical extraction. At 24 h, Mn existed both inside and outside the cells, mainly in the forms of extracellularly and intracellularly adsorbed fractions (Figure 3A). The amount of Mn adsorbed extracellularly at 24 h ranged from 0.01 to 0.88 mM, while the amount adsorbed intracellularly ranged from 0.03 to 0.32 mM. At this time point, the amount of oxidised Mn was relatively low; the highest oxide content was obtained in the treatment with an initial Mn concentration of 2 mM, reaching 0.59 mM. At 72 h of cultivation, the residual soluble Mn concentration in the supernatant decreased sharply, while the adsorbed fractions remained low and the oxidised fraction increased sharply (Figure 3B). In the 5 mM treatment, the oxidised Mn fraction (the hydroxylamine-reducible solid fraction, F4) reached 3.42 mM at 72 h, and the total Mn recovered across all four fractions was 4.18 mM, corresponding to an 83.6% recovery of the initial 5.0 mM Mn(II). Approximately 95% of the initial Mn(II) was removed from the solution phase (residual soluble Mn = 0.248 mM at 72 h) through adsorption and oxidation. It should be noted that the LBB-based measurement in Section 3.2 and the AAS-based measurements here are not directly comparable because they quantify different operational fractions and were obtained at different time points. LBB specifically detects the Mn(III)/Mn(IV) oxide pool, whereas the oxidised fraction determined by AAS represents the total Mn released by acidic hydroxylamine hydrochloride, which may also dissolve the co-precipitated MnCO_3_ identified by XRD and XPS. Across all treatments and time points, the recovery of Mn (the sum of the four fractions relative to the initial Mn(II)) ranged from 71.2 to 113.2% (Supplementary Table S2). A complete mass balance of the Mn species under these conditions is provided in Supplementary Table S2.

**Figure 3:**
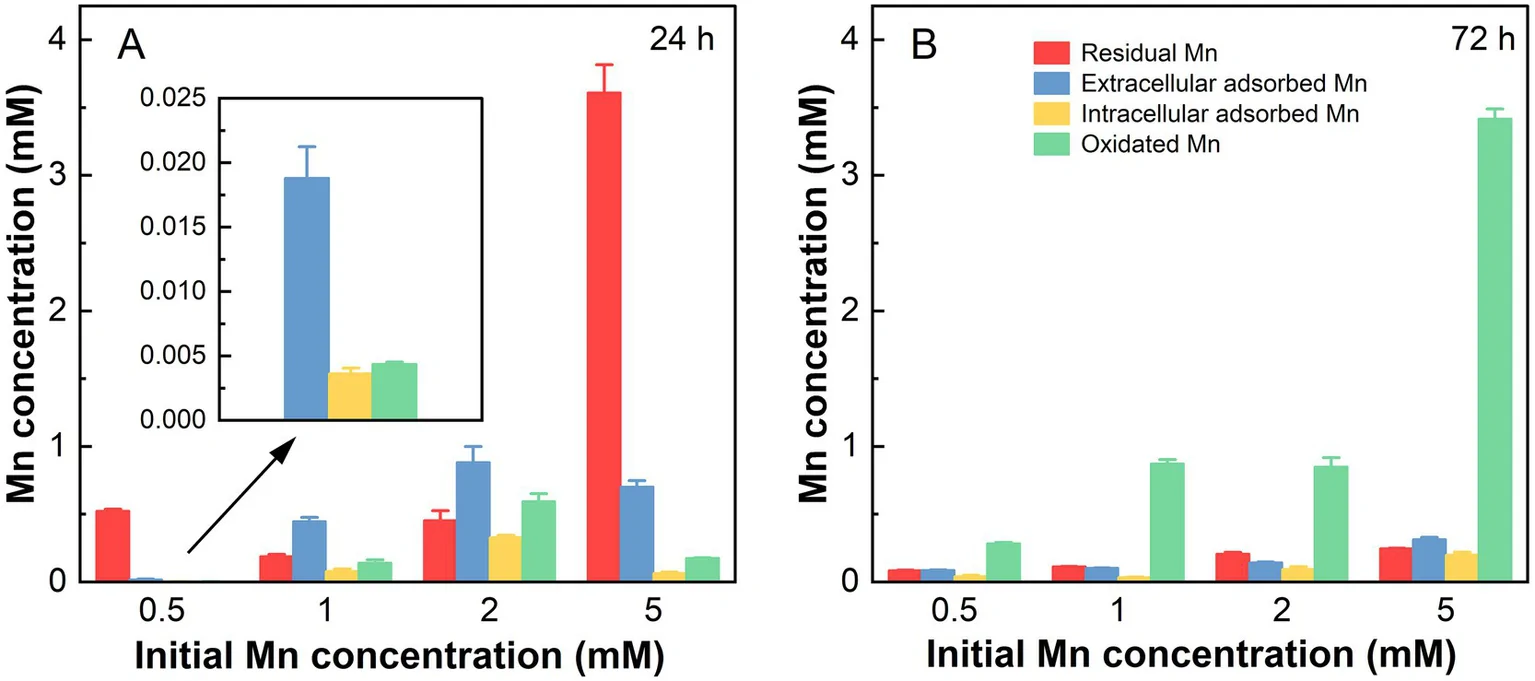
Subcellular localisation of bacterial manganese oxidation at 24 h **(A)** and 72 h **(B)**.

### 3.4 Mechanisms of manganese bio-oxidation by strain DX3

#### 3.4.1 ROS generation during Mn(II) oxidation by strain DX3

Reactive oxygen species (ROS) possess strong oxidising properties and may participate in the redox transformations of metal elements. To examine the potential contribution of ROS to Mn(II) oxidation by strain DX3, the concentrations of hydroxyl radical (·OH), superoxide anion (O₂^−^·), and hydrogen peroxide (H₂O₂) in the cultures were determined. The OH concentration increased with increasing incubation time and initial Mn^2+^ concentrations (Figure 4A). Notably, this pattern paralleled the increase in biogenic manganese oxide (BMO) formation with increasing initial Mn^2+^ concentrations (Figure 2D), suggesting a possible association between ·OH production and Mn(II) oxidation. In contrast, the O₂^−^· concentration decreased with increasing incubation time, and no clear correlation with the initial Mn^2+^ concentration was observed (Figure 4B). The H₂O₂ concentration reached its maximum at 24 h and then decreased sharply by 72 h (Figure 4C); at 24 h, H₂O₂ tended to increase with rising initial Mn^2+^ concentrations, whereas no obvious trend was observed at 48 h. It should be emphasised that these results establish correlations only and do not demonstrate that ROS directly mediate Mn(II) oxidation. In Mn(II)-oxidising bacteria, direct enzymatic oxidation (e.g., by multicopper oxidases) is generally considered the primary pathway, and the observed ROS may partly reflect the cellular oxidative stress response induced by elevated Mn(II) concentrations.

**Figure 4:**
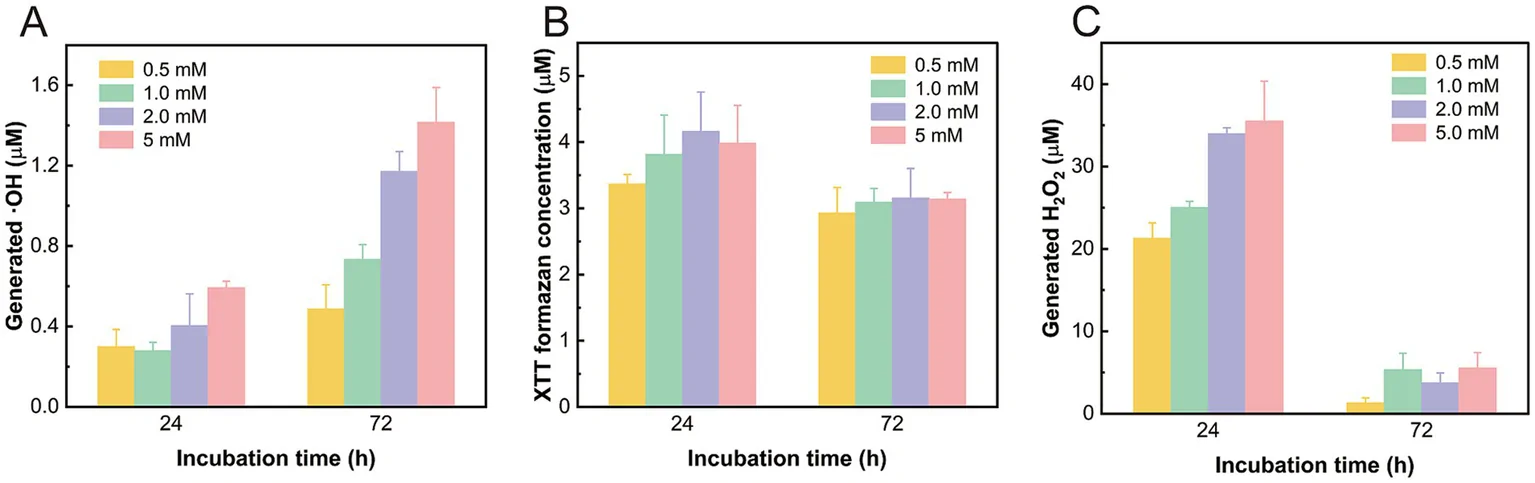
Changes in reactive oxygen species concentrations in the culture medium at different incubation time intervals. Hydroxyl radical production **(A)**; superoxide radical production **(B)**; and hydrogen peroxide production **(C)**. Data are presented as mean ± SD (*n* = 3).

#### 3.4.2 Mechanisms of manganese bio-oxidation by strain DX3

It has been reported that Mn(III) act as an intermediate in microbial Mn(II) oxidation. Unstable Mn(III) could react quickly and form manganese oxides. To investigate the role of Mn(III) in Mn(II) oxidation mediated by strain DX3, PP was employed to trap potential Mn(III). The concentration of generated BMOs was completely inhibited in the cultivation group with PP added, while the group without PP showed normal trends in BMO production (Figure 5A). These results indicate that Mn(II) was first oxidised to Mn(III) by strain DX3. Mn(III) acted as an intermediate during cultivation. A slight increase in BMO concentration was observed after 4 d of incubation, which might be attributed to the decay of Mn(III)–PP complexes, as also reported by Li et al. (2021) and Yan et al. (2019). The UV–visible spectra of the cultivation medium at different incubation times support this inference (Figure 5B). The absorbance of the samples with PP addition increased continuously, reaching a maximum on day 4 and then decreasing sharply on day 10, indicating a decrease in Mn(III)–PP complexes.

**Figure 5:**
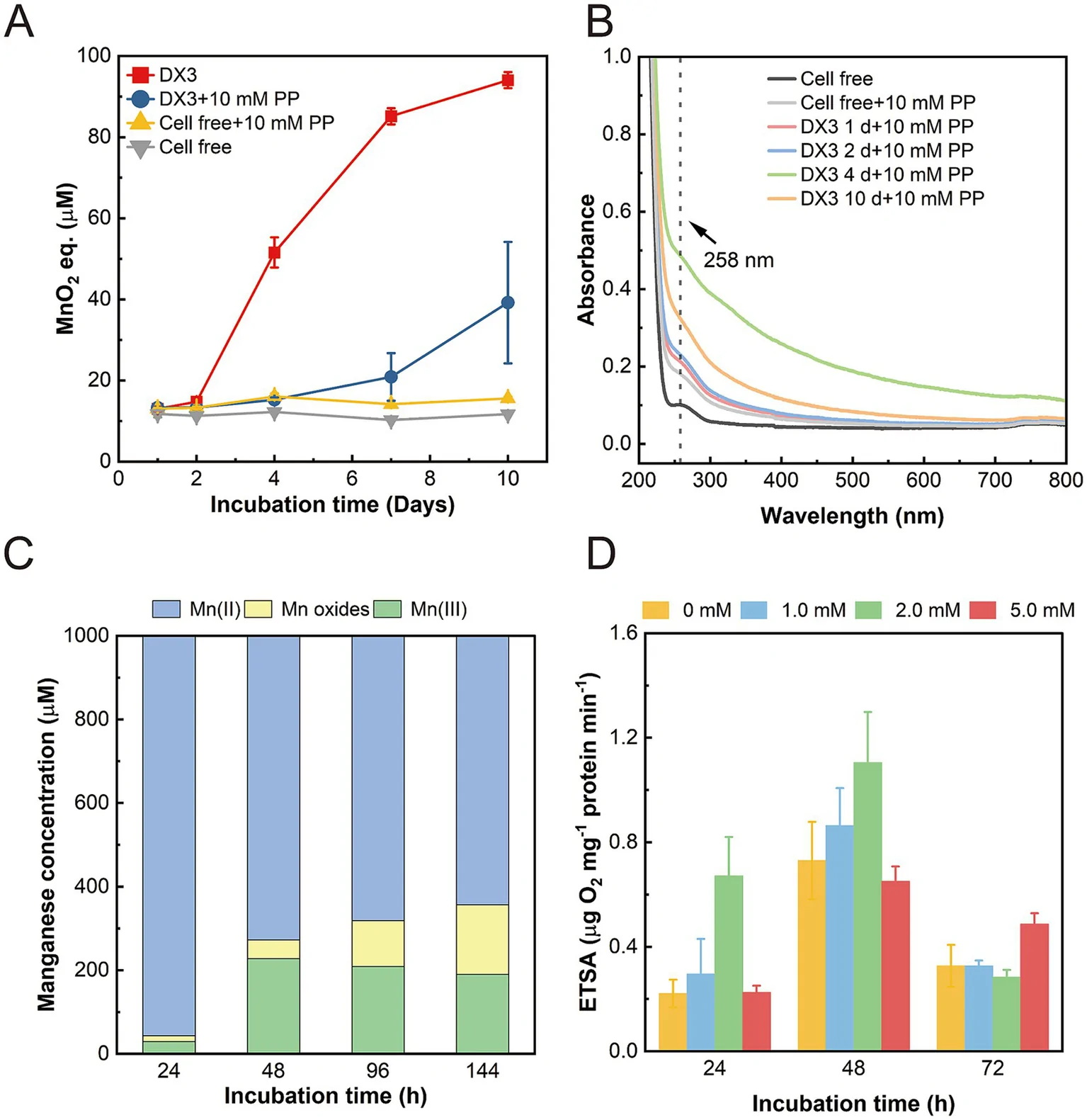
Manganese oxide generation during strain cultivation with or without PP addition **(A)**. UV–visible spectra of the suspension at different times **(B)**. Manganese fractions during the cultivation of strain DX3 **(C)**. ETSA at different incubation times and initial Mn concentrations **(D)**. Data are presented as mean ± SD (*n* = 3).

To further characterise changes in manganese speciation during cultivation of strain DX3, we measured the concentrations of soluble Mn(II) and Mn(III) and the content of Mn oxides (Figure 5C). After 24 h of incubation, only small amounts of Mn(III) and Mn oxides were found in the cultivation medium. The concentrations of Mn(III) and Mn oxides increased to 227.9 μM and 44.64 μM, respectively. The concentration of Mn oxides increased continuously at 96 h and 144 h.

To further elucidate the effect of manganese concentration on BMO production by strain DX3, we measured electron transport system activity (ETSA) after strain cultivation with different concentrations of Mn(II) at 24 h, 48 h, and 72 h (Figure 5D). It can be observed that as the initial Mn(II) concentration increased from 0 to 2 mM, ETSA increased from 0.22 to 0.67 μg O_2_ mg^−1^ protein min^−1^ at 24 h and from 0.73 to 1.10 μg O_2_ mg^−1^ protein min^−1^ at 48 h. However, ETSA decreased when the initial Mn(II) concentration was 5 mM. In addition, ETSA at 48 h was much higher than that at 24 h, suggesting enhanced electron transfer activity during Mn(II) oxidation by strain DX3. Moreover, ETSA decreased sharply at 72 h, which may be attributed to the slower growth and metabolism of strain DX3.

#### 3.4.3 Characterisation of biogenic manganese oxides

The workflow of BMO preparation and characterisation is illustrated in Figure 6A. The TEM image showed that the BMOs generated by DX3 had an irregular, loose, layered structure (Figures 6B,C). Flake-shaped and porous structures were also observed in the image. The selected area electron diffraction (SAED) pattern indicated the poor crystallinity of the BMOs, consistent with previous studies reporting BMOs generated by manganese-oxidising bacteria (Figure 6D) (Zhang et al., 2022a). The XRD pattern of the BMOs formed by strain DX3 after 7 d of incubation with 5 mM Mn(II) showed a few weak, broad reflections at 2θ ≈ 22°, 31° and 33° (Figure 6E). These reflections were most consistent with poorly crystalline MnO_2_, and Mn_2_O_3_, with a minor MnCO_3_, phase, consistent with previous reports Zhang et al. (2024). The XRD diffraction patterns of MnO_2_ and Mn_2_O_3_ showed broad, amorphous features and no peaks indicative of crystalline phases. In contrast, the diffraction peaks of MnCO_3_ were sharp and intense, indicating its highly crystalline nature.

**Figure 6:**
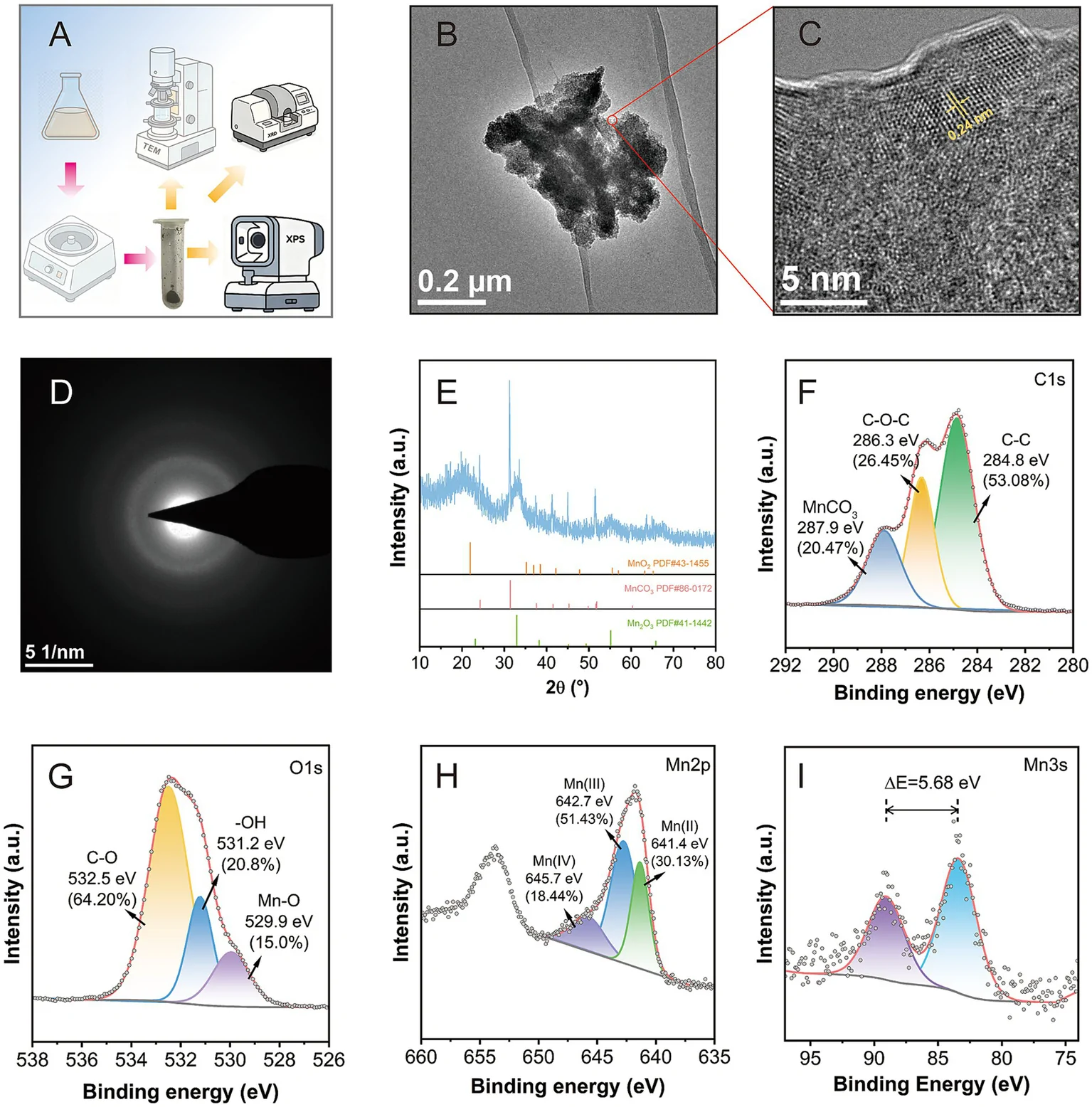
Characterisation of the biogenic manganese oxides (BMOs) generated by strain DX3. **(A)** Schematic diagram of BMO preparation and characterisation; **(B,C)** TEM images; **(D)** SAED pattern; **(E)** XRD pattern; and **(F–I)** XPS spectra of C 1 s, O 1 s, Mn 2p, and Mn 3 s, respectively.

XPS was employed to investigate changes in elements on the surface of biogenic manganese oxides generated by strain DX3. Peaks assigned to C1s, O1s, Mn2p, and Mn3s were observed in the full-scan spectrum of the biogenic manganese oxides (Supplementary Figure S5). The fitting lines displayed three peaks centred at 287.9, 286.3, and 284.8 eV in the C1s region (Figure 6F). The peaks centred at 287.9 and 286.3 eV were assigned to MnCO_3_ and C-O-C, respectively. The peaks assigned to MnCO_3_ were consistent with the diffraction results, demonstrating the formation of MnCO_3_ during the cultivation of DX3. The peaks centred at 532.5, 531.2, and 529.9 eV in the O 1 s region were assigned to C-O, -OH, and Mn-O, respectively (Figure 6G) (Hou et al., 2020; Rajaram et al., 2023). The peaks corresponding to C-O may be attributed to the formation of MnCO_3_. Meanwhile, the -OH peaks could be attributed to manganese hydroxides generated during bacterially mediated manganese oxidation. The peaks at 529.9 eV were typically Mn-O, indicating the formation of manganese oxides. The peaks centred at 645.7, 642.7, and 641.4 eV were assigned to Mn(IV), Mn(III), and Mn(II), respectively (Figure 6H) (Wang et al., 2024; Ma et al., 2023). Mn(II) may be the adsorbed cation on the surface of BMOs, which have a relatively large specific surface area and high adsorption capacity. The XPS spectrum of the Mn 2p_3/2_ region showed that Mn(III) was generated during microbial oxidation. During cultivation, Mn(III) may exist in biogenic manganese oxides as trivalent oxides or hydroxides. In addition, the binding energy difference between the centres of the two peaks in the Mn 3 s region of the XPS spectrum was 5.68 eV, indicating the chemical state of Mn(III) (Figure 6I).

## 4 Discussion

The oxidation of Mn(II) in the medium cultivating strain DX3 was strongly correlated with the biomass growth of strain DX3, indicating that DX3 was dominant in the oxidation process. In this study, no evident BMO precipitates were detected in the culture flask without strain DX3 inoculation. This phenomenon has also been observed by other researchers, proving that Mn(II) oxidation proceeds very slowly in the presence of only natural oxygen (Zhang et al., 2022b; Feng et al., 2024). In addition, the amount of BMO precipitates increased rapidly with prolonged cultivation. The trend of BMO precipitate accumulation was correlated with the growth curve of strain DX3. Notably, in the first 48 h, very few BMO precipitates were generated, which differed little from the group without strain DX3 inoculation. This could mainly be attributed to the strain still being in the initial growth phase, with more Mn(II)-oxidising substances yet to be secreted (Li et al., 2024). Collectively, this evidence demonstrates that strain DX3 has Mn(II)-oxidising activity and that Mn(II) oxidation in this study was mediated by the growth of strain DX3 (Figure 7).

**Figure 7:**
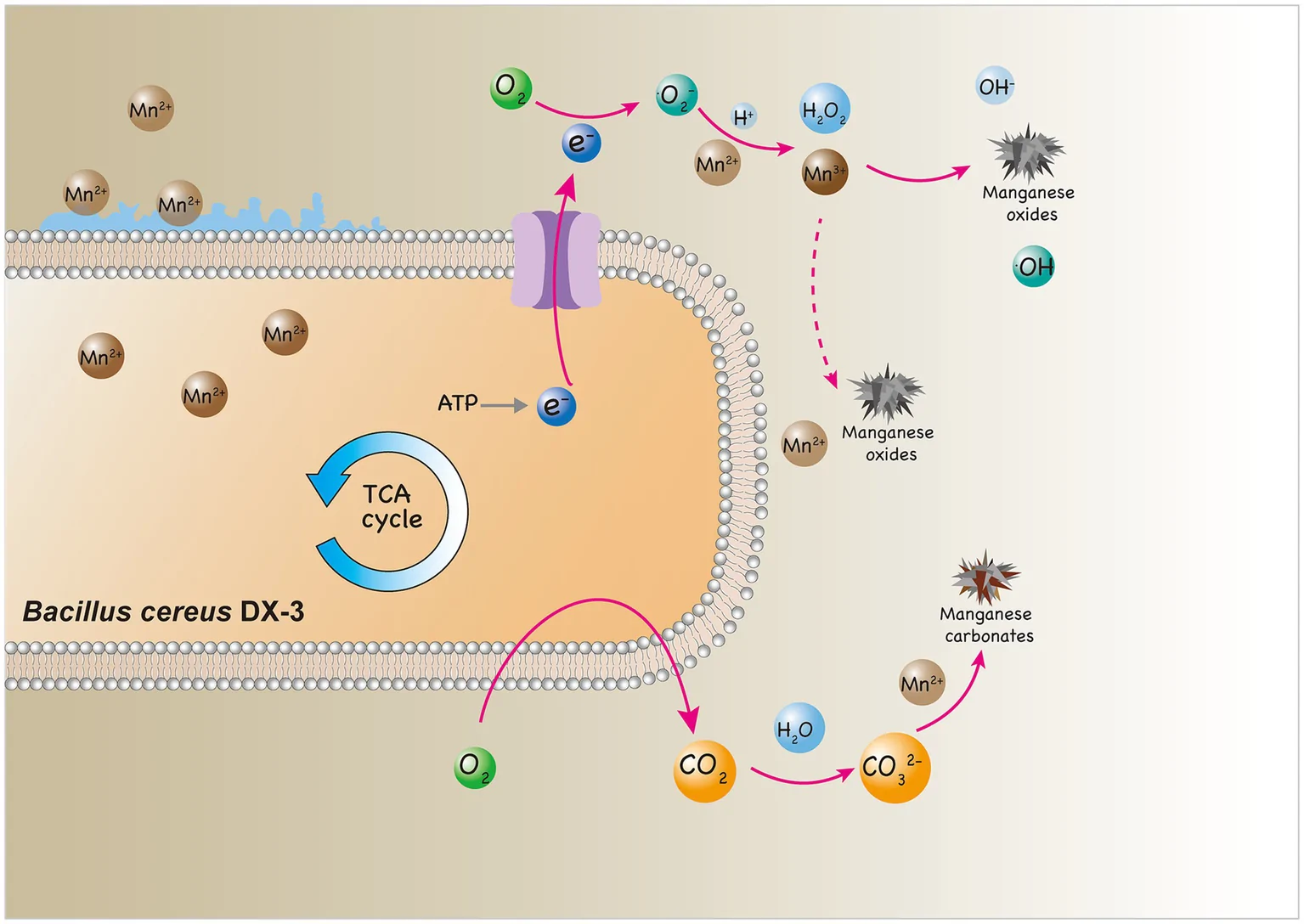
Schematic diagram of Mn^2+^ oxidation by strain DX3.

Biological Mn(II) oxidation is generally linked to both intracellular and extracellular electron transfer. To further explore the possible role of Mn(II) in the electron transfer system of strain DX3, we determined the electron transfer system activity of strain DX3 cultivated with different initial concentrations of Mn(II). Initial Mn(II) concentrations in the range of 0–2 mM effectively enhanced the electron transfer system activity of DX3. This may be attributed to the response of DX3 cells to Mn(II)-induced oxidative stress (Martínez-Ruiz et al., 2021). Previous studies have shown that manganese-oxidising bacteria could upregulate the expression of manganese oxidising-related genes, thereby accelerating the secretion of manganese-oxidising enzymes (Kumar and Arora, 2022). As intermediates, manganese-oxidising enzymes could effectively facilitate electron transfer between oxygen and Mn(II). However, ETSA decreased when the initial Mn(II) concentration was 5 mM, which could mainly be attributed to the inhibition of Mn(II) on cell respiration.

The oxidation of Mn(II) to Mn(III)/Mn(IV) oxides is accompanied by electron transfer. Theoretically, Mn(II) could be oxidised to Mn(III) and then further oxidised to Mn(IV) by sequentially donating one electron, or it can be directly oxidised to Mn(IV) by donating two electrons. In the sequential oxidation pathway, Mn(III) plays an important role as an intermediate in the transformation of Mn(II) to Mn(IV). In this study, the addition of PP largely reduced the generation of biogenic manganese oxide precipitates, indicating that Mn(III) acted as an intermediate during Mn(II) oxidation by strain DX3. Similar findings have also been reported by Bai et al. Bai et al. (2021). In addition, the UV–visible spectra of the strain DX3 cultures at different times showed that Mn(II) was initially oxidised to Mn(III) and subsequently to Mn(IV) during oxidation. The generation of precipitated manganese oxides requires the accumulation of soluble Mn(III). These results also explain the observation that the concentration of precipitated manganese oxides remained relatively low during the first 48 h of cultivation.

Previous studies have shown that the main components of biogenic manganese oxides are MnO_2_, Mn_2_O_3_, and MnO. The XRD results showed that the precipitates were formed by MnO_2_, Mn_2_O_3_, and MnCO_3_. The formation of Mn(III)/Mn(IV) oxides corresponded with the results of the Mn 2p and Mn 3 s XPS analyses. Interestingly, the cultivation of DX3 generated MnCO_3_, which is a carbonate precipitate with high crystallinity. The formation of MnCO_3_ may be attributed to the generation of bicarbonate and carbonate during cultivation. Aerobic respiration of microbes releases CO_2_, which then dissolves in the medium (Ma et al., 2023). The generated carbonate species in the culture medium immediately combined with Mn^2+^and formed MnCO_3_ precipitates. In addition, the formation of MnCO_3_ might also be attributed to organic nitrogen decomposition and ammonium generation. Similar results have also been reported in studies of microbiologically induced calcium carbonate precipitation (MICP) (Ma et al., 2024). Based on the results of XPS, the formation of MnCO_3_ was identified on the surface of biogenic manganese oxide precipitates generated by strain DX3. In addition, three valences of manganese [Mn(II), Mn(III), and Mn(IV)] were also identified. The Mn(II) identified in the solid fraction may be the residual Mn(II) adsorbed on the surfaces of the generated BMOs. The Mn(III) detected might arise from surface-adsorbed Mn(III), ligand-bound Mn(III), or the Mn_2_O_3_ present in the precipitate fraction. Notably, Mn(IV) accounted for only 18.44% of the total manganese signal, implying that Mn(IV) was formed through the further oxidation of Mn(III). The average oxidation state of the precipitates showed that Mn(III) constituted the dominant proportion of the oxidised manganese.

Biogenic manganese oxides (BMOs) contribute to the remediation of metal-contaminated environments through three interrelated mechanisms. First, BMOs are strong adsorbents and scavengers of trace metals: The poorly crystalline, high-surface-area manganese oxides produced by Mn(II)-oxidising bacteria efficiently adsorb Pb, Zn, Cu, Ni, and Cd through surface complexation and coprecipitation (Tebo et al., 2004; Wang and Chen, 2009), thereby immobilising these metals in soils and aquatic environments. Second, Mn(III)/Mn(IV) oxides are strong oxidants that can transform redox-sensitive contaminants. For example, Mn oxides can oxidise the more toxic and mobile As(III) to the less toxic and more strongly adsorbing As(V) (Nelson and Lion, 2003; Remucal and Ginder-Vogel, 2014) and can also oxidatively degrade organic contaminants such as diclofenac (Huguet et al., 2013; Grebel et al., 2016). Third, Mn(II) oxidation activity itself removes dissolved Mn(II)—the target contaminant—by converting it into solid BMOs, which can then be separated or immobilised (Wang and Chen, 2009; Kitjanukit et al., 2017). In the present study, strain DX3 produced BMOs with a mixed MnO2/Mn2O3/MnCO3 composition, and the Mn(II) → Mn(III) → Mn(IV) pathway identified here is directly relevant to these remediation mechanisms, as it is the Mn(III)/Mn(IV) oxide products that confer the adsorptive and oxidative functions described above. Nevertheless, as discussed in the Conclusion, the practical efficacy of strain DX3 in real-world soil and water matrices remains to be validated.

## 5 Conclusion

In this study, the manganese-oxidising bacterium *Bacillus wiedmannii* DX3 was successfully isolated from electrolytic manganese residue and demonstrated efficient Mn(II) oxidation under aerobic conditions. The maximum biogenic manganese oxide production reached 166.89 μM at an initial Mn(II) concentration of 5.0 mM. Spectroscopic and chemical analyses confirmed that Mn(III) serves as a critical intermediate during the microbial oxidation process, with pyrophosphate trapping experiments further validating the sequential oxidation pathway of Mn(II) → Mn(III) → Mn(IV). Characterisation of the biogenic manganese oxides revealed a mixed composition of MnO_2_, Mn_2_O_3_, and MnCO_3_, as determined by XRD, TEM, and XPS. Electron transfer system activity assays indicated that appropriate Mn(II) concentrations (0–2 mM) promote microbial electron transfer, whereas higher concentrations (5 mM) exhibit inhibitory effects. In addition, the generation of reactive oxygen species, particularly ·OH, was found to correlate positively with Mn(II) concentration and incubation time, suggesting their potential role in the oxidation process. Collectively, these findings elucidate the manganese oxidation mechanism of *Bacillus wiedmannii* DX3 and demonstrate how the resulting BMOs can underpin bioremediation: By immobilising toxic metals through adsorption and coprecipitation, by oxidising As(III) and organic contaminants, and by directly removing dissolved Mn(II) as a solid oxide phase. However, the efficacy of strain DX3 in real-world soil and water matrices, and the biosafety of this *B. wiedmannii* strain, require further evaluation before any practical application can be considered.

## Data Availability

The original contributions presented in the study are included in the article/Supplementary material, further inquiries can be directed to the corresponding author.
