# Supplementary material for: Manganese(II) oxidation by Bacillus wiedmannii DX3 isolated from electrolytic manganese residue: Mn(III) as an intermediate and characterisation of biogenic manganese oxides
Source: Front Microbiol. 2026 Oct 13;17:1910017. doi: 10.3389/fmicb.2026.1910017 (PMC13710301; doi:10.3389/fmicb.2026.1910017)
Supplement: Supplementary file 1 [file Supplementary_file_1.docx]

Supplementary information

# Manganese(II) oxidation by *Bacillus wiedmannii* DX3 isolated from electrolytic manganese residue: Mn(III) intermediate and characterization of biogenic manganese oxides

Kai Zhong^a^, Qian Zhang^a^, Siting Lu^b^, Juanjuan Yin^b^, Yan Liao^b^, Minghua Gu^a^, Chaolan Zhang^a,b*^

^a^ College of Agriculture, Guangxi University, Nanning, 530004, China

^b^ School of Resource, Environment, and Materials, Guangxi University, Nanning, 530004, China

^*^ Corresponding author. College of Agriculture, Guangxi University, Nanning, 530004, China. E-mail address: zhangcl@gxu.edu.cn

Summary: 9 pages, 1 text, 1 table, and 6 figures.

**Supplementary text**

Culture medium

LB medium (per litre): 5 g yeast extract, 10 g NaCl, 10 g peptone.

PYG medium (per litre): 0.25 g peptone, 0.25 g yeast extract, 0.25 g glucose, 0.6 g CaCl_2_, 0.06 g MgSO_4_. Autoclaved at 115 °C for 30 min. Mn^2+^was added into the medium as MnCl_2_, after filtered by sterile 0.22 μm membrane.

**Supplementary table**

Supplementary Table S1 Fitting parameters of XPS spectra

| Lines | Binding energy (eV) | Line designation | % |
| --- | --- | --- | --- |
| C1s | 284.8 | C-C | 53.08 |
|  | 286.3 | C-O-C | 26.45 |
|  | 287.9 | MnCO_3_ | 20.47 |
| O1s | 529.9 | Mn-O | 15.00 |
|  | 531.2 | -OH | 20.80 |
|  | 532.5 | C-O | 64.20 |
| Mn2p_2/3_ | 641.4 | Mn(II) | 30.13 |
|  | 642.7 | Mn(III) | 54.13 |
|  | 645.7 | Mn(IV) | 18.44 |

Supplementary Table S2 Distribution and recovery of manganese during Mn(II) oxidation by strain DX3.

|  | Initial Mn^2+^ concentration (mM) | F1 (mM) | F2 (mM) | F3 (mM) | F4 (mM) | sum (mM) | recovery (%) |
| --- | --- | --- | --- | --- | --- | --- | --- |
| 24 h | 0.5 | 0.525 ± 0.012 | 0.019 ± 0.002 | 0.004 ± 0.000 | 0.004 ± 0.000 | 0.552 | 110.425 |
|  | 1 | 0.190 ± 0.015 | 0.448 ± 0.029 | 0.080 ± 0.016 | 0.142 ± 0.022 | 0.859 | 85.936 |
|  | 2 | 0.456 ± 0.070 | 0.883 ± 0.117 | 0.329 ± 0.015 | 0.596 ± 0.056 | 2.264 | 113.202 |
|  | 5 | 3.611 ± 0.207 | 0.703 ± 0.045 | 0.066 ± 0.007 | 0.176 ± 0.003 | 4.555 | 91.105 |
| 72 h | 0.5 | 0.086 ± 0.002 | 0.088 ± 0.002 | 0.041 ± 0.008 | 0.284 ± 0.008 | 0.499 | 99.879 |
|  | 1 | 0.115 ± 0.000 | 0.103 ± 0.001 | 0.035 ± 0.002 | 0.873 ± 0.029 | 1.126 | 112.628 |
|  | 2 | 0.208 ± 0.011 | 0.144 ± 0.003 | 0.101 ± 0.017 | 0.971 ± 0.068 | 1.424 | 71.200 |
|  | 5 | 0.248 ± 0.002 | 0.315 ± 0.014 | 0.201 ± 0.019 | 3.418 ± 0.072 | 4.182 | 83.642 |

Note: F1, residual Mn; F2, extracellular adsorbed Mn; F3, intracellular adsorbed Mn; F4, oxidised Mn

**Supplementary figures**

Supplementary Figure S1 Gram staining result image of strain DX3.

Supplementary Figure S2 Calibration curve of manganese oxide determination by LBB assay

Supplementary Figure S3 The colony morphology of the manganese-oxidising bacteria DX-3 on agar plate with different Mn^2+^ concentrations.

Supplementary Figure S4 Distribution of Mn(II) under different pH regimes

Supplementary Figure S5 Full scan spectrum of biogenic manganese oxides generated by strain DX-3 after 7 d incubation.

Supplementary Figure S6 Changes of Mn(III) concentration (A) and manganese oxides generation (B) with different initial Mn^2+^ concentrations after 24 and 72 h incubation.

Supplementary Figure S7 Manganese oxides generation of strain DX-3 with or without H_2_O_2_ addition after 72 h incubation
